# Current methods of sedation in dental patients - a systematic review of the literature

**DOI:** 10.4317/medoral.20981

**Published:** 2016-07-31

**Authors:** Jose-Ramón Corcuera-Flores, Javier Silvestre-Rangil, Antonio Cutando-Soriano, Julián López-Jiménez

**Affiliations:** 1 PhD, DDS, Associate Professor. Special Care in Dentistry, School of Dentistry, University of Seville, Spain; 2 PhD, DDS, Associate Professor. Special Care in Dentistry, School of Dentistry, University of Valencia, Spain; 3 PhD, MD, DDS, Professor. Special Care in Dentistry, School of Dentistry, University of Granada, Spain; 4 PhD, MD, DDS. “Nen Deu” Private Practice Hospital, Barcelona, Spain

## Abstract

**Objetive:**

The main objective of this systematic literature review is to identify the safest and most effective sedative drugs so as to ensure successful sedation with as few complications as possible.

**Study Design:**

A systematic literature review of the PubMed MEDLINE database was carried out using the key words “conscious sedation,” “drugs,” and “dentistry.” A total of 1,827 scientific articles were found, and these were narrowed down to 473 articles after applying inclusion and exclusion criteria. These 473 studies were then individually assessed for their suitability for inclusion in this literature review.

**Results:**

A total of 21 studies were selected due to their rigorous study design and conduciveness to further, more exhaustive analysis. The selected studies included a total of 1,0003 patients classified as ASA I or II. Midazolam was the drug most frequently used for successful sedation in dental surgical procedures. Ketamine also proved very useful when administered intranasally, although some side effects were observed when delivered via other routes of administration. Both propofol and nitrous oxide (N2O) are also effective sedative drugs.

**Conclusions:**

Midazolam is the drug most commonly used to induce moderate sedation in dental surgical procedures, and it is also very safe. Other sedative drugs like ketamine, dexmedetomidine and propofol have also been proven safe and effective; however, further comparative clinical studies are needed to better demonstrate which of these are the safest and most effective.

**Key words:**Conscious sedation, drugs, dentistry.

## Introduction

Conscious sedation is an effective method of reducing preoperative anxiety in children and in adult patients who suffer from anxiety, especially prior to surgical procedures requiring general anesthesia. When administered before dental treatments, conscious sedation methods have been shown to aid in the reduction of patient pain and anxiety. Conscious sedation is very useful in encouraging patient cooperation and improving overall patient satisfaction with dental treatment. However, conscious sedation methods do involve some level of risk for patients and dental practitioners (1). It is well known that conscious sedation allows dental practitioners to treat uncooperative patients (2).

Some patients simply cannot be treated with loco regional anesthesia alone for various reasons, generally due to behavioral problems resulting from some form of disability or because the patient is a child. In these cases, procedures must be performed with the patient under conscious sedation (3). However, in some cases requiring very complex dental procedures, or if the patient is in poor condition, conscious sedation may be inadvisable or the class of drugs used may be contraindicated. The adverse effects associated with conscious sedation are a result of the class of drugs used, with hallucinations being the most frequently observed adverse reaction (4,5) linked to the use of benzodiazepines, propofol and nitrous oxide. Nitrous oxide may also cause damage to immune and hematologic systems, and it can cause fertility problems in women (6-9). However, the biggest disadvantage of conscious sedation is that it can mask symptoms of a medical emergency, so clinicians should remain very conscious of proper methods of sedation for dental procedures and their importance (10).

Clinics that employ methods of conscious sedation are required to have the equipment necessary to handle medical emergencies such as hypoventilation or central nervous system depression (11-13). The most important consideration when dealing with a potential emergency is to have a highly qualified team capable of handling any issues that may arise, especially any respiratory complications.

Today, there are a wide variety of drugs that can be used to sedate patients (14); however, there are relatively few studies that compare the safety and effectiveness of different kinds of sedatives. Therefore, the main objective of this systematic literature review is to identify the safest and most effective sedative drugs so as to ensure successful sedation with as few complications as possible.

## Material and Methods

To fulfill the given objectives, a systematic literature review was undertaken using the PubMed MEDLINE database, with a view to identifying the safest and most effective sedative drug in order to provide dental practitioners with updated information on whichever drugs were found to be the most suitable. A total of 4,740 scientific articles were found by entering the key words “drugs” and “sedation” into the PubMed MEDLINE database. The search was then further limited to clinical trials, which narrowed the results down to 473 studies.

These 473 results were then individually assessed for their suitability for inclusion in this literature review, with a total of 21 studies being selected due to their rigorous study design and conduciveness to further, more exhaustive analysis; in this case, only prospective randomized studies were classified as rigorous.

**Figure 1:**
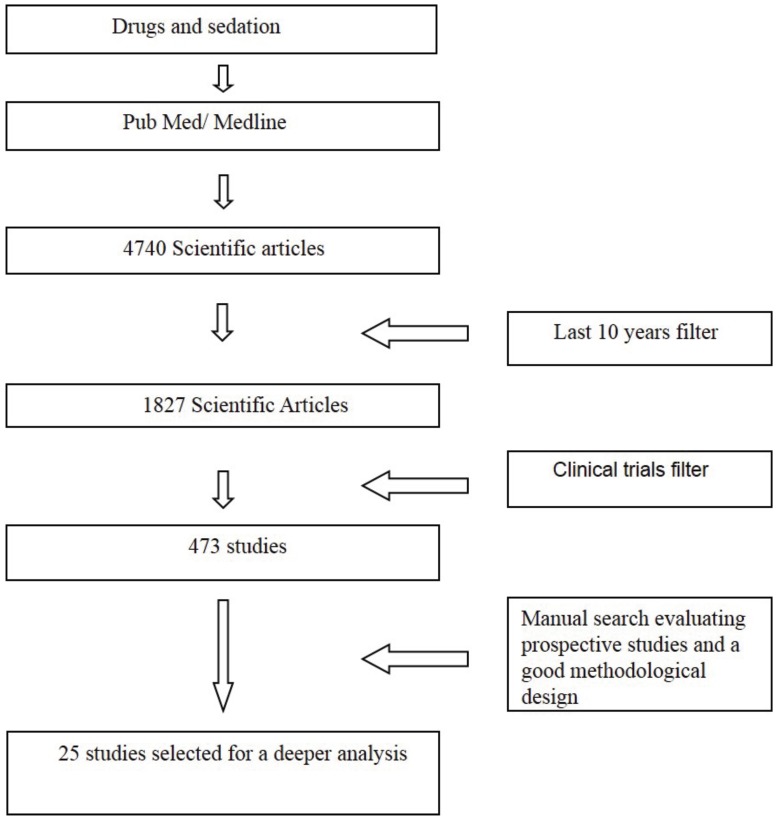
Literature Review Diagram.

The only studies selected were prospective randomized studies; any studies that were not prospective were discarded. Other inclusion criteria stipulated that studies focus on sedative drugs administered to either healthy patients or patients with specialized treatment needs, including need for buccal or cervicofacial surgical intervention, or studies that compared and assessed different drugs used to induce light or moderate sedation. See figure 1 for a diagram detailing how this literature review was carried out.

## Results

The selected articles studied a total of 1,003 patients classified as ASA I or II.

Table  Table 1 and  Table 1continue provides an overview of each of the selected articles: Authors, year of publication, number of patients treated, drugs administered, route(s) of administration, medical specialty, and conclusions reached.

**Table 1:**
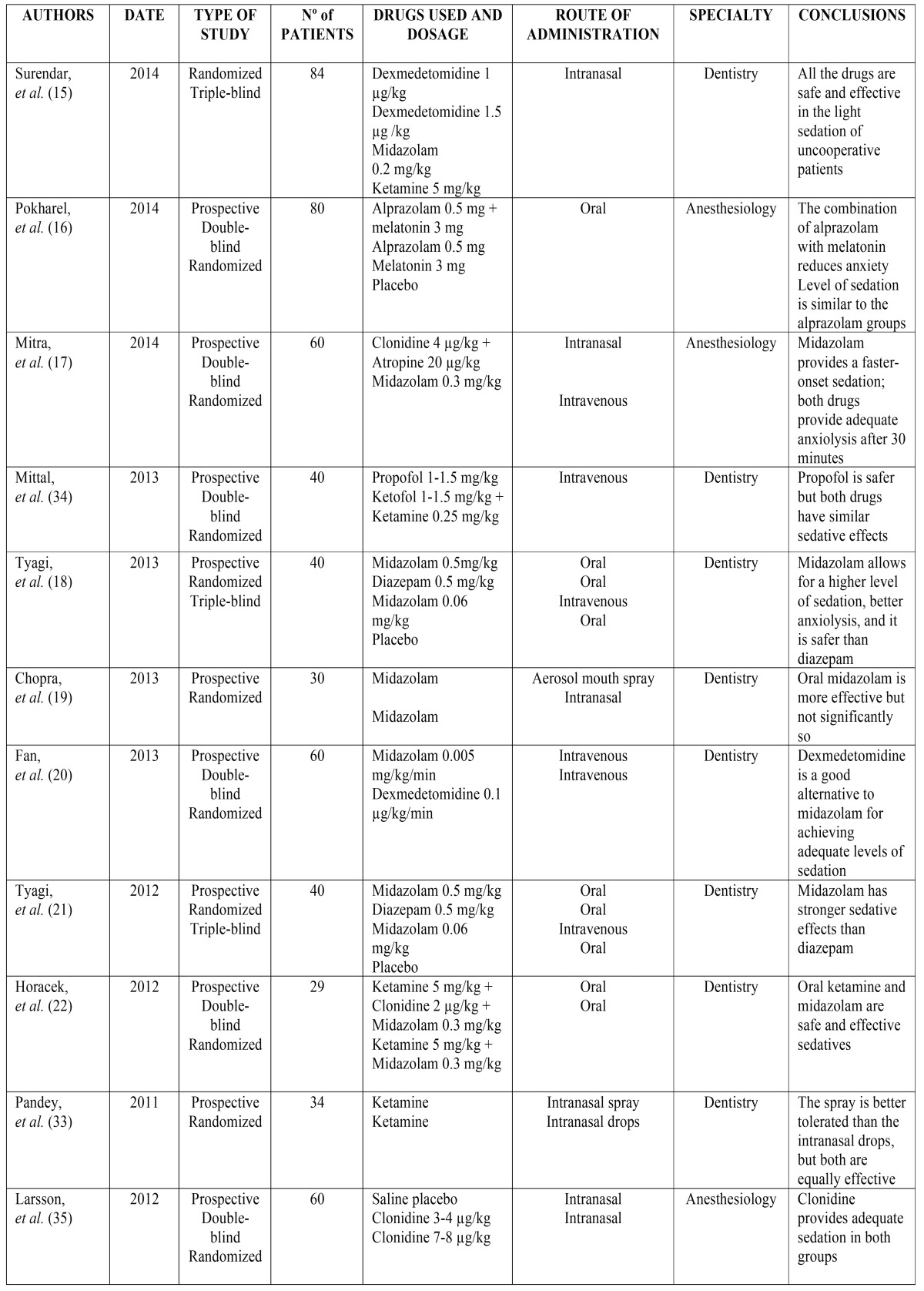
Authors, year of publication, number of patients, drugs administered, route of administration, medical specialty, and conclusions of each of the analyzed articles.

**Table 1 continue:**
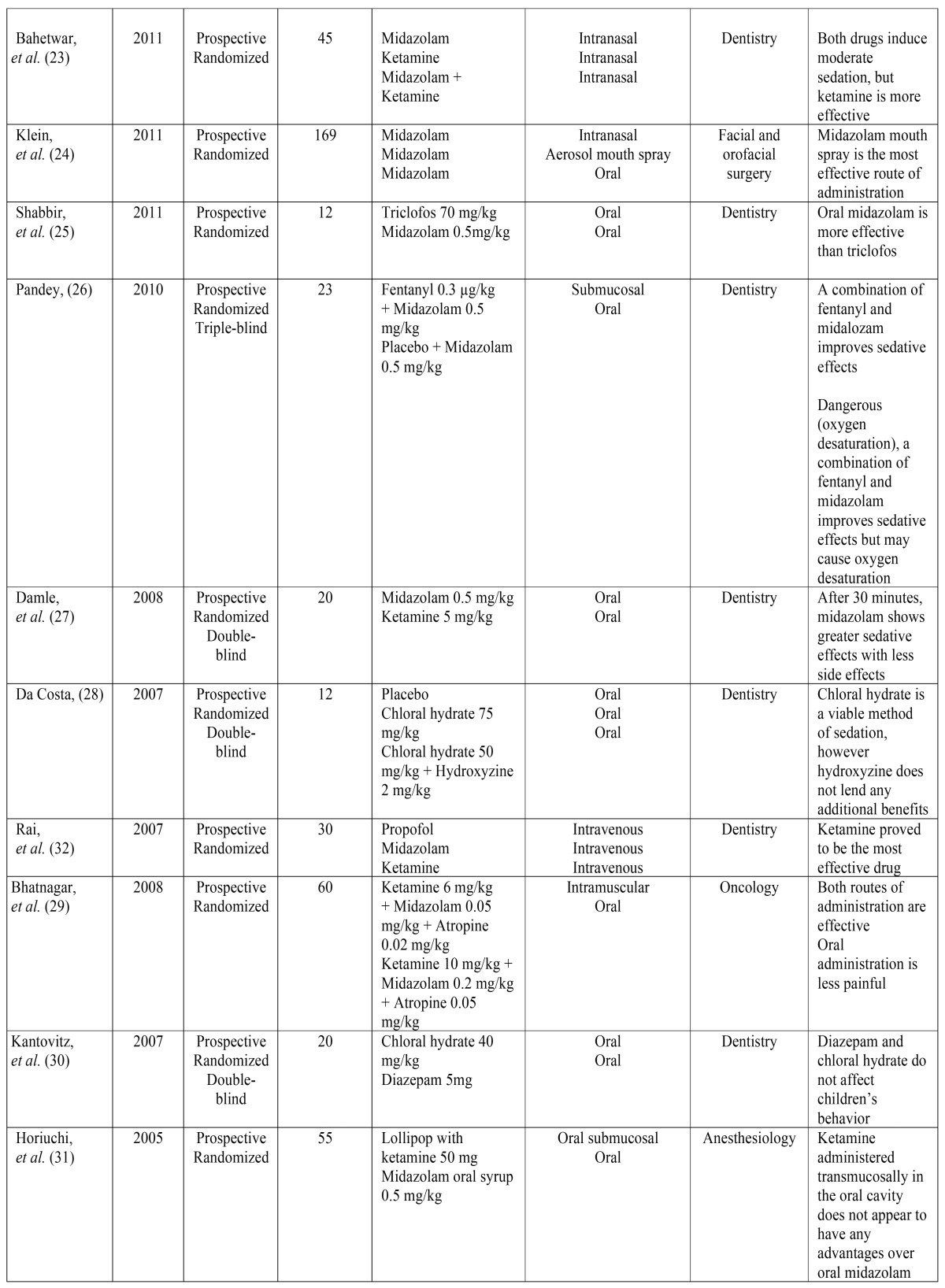
Authors, year of publication, number of patients, drugs administered, route of administration, medical specialty, and conclusions of each of the analyzed articles.

Upon analyzing the different kinds of sedative drugs used, it appears midazolam was used 24 times in 15 studies, in varying concentrations (0.005 mg/kg, 0.06 mg/kg, 0.2 mg/kg, 0.3 mg/kg, and 0.5 mg/kg). It was administered orally in 10 studies, intranasally in 5, intravenously in another 5 studies, and in one study it was administered via an aerosolized buccal spray. On one occasion, it was administered using a transmucosal syrup, through intramuscular injection, and submucosally. All of the studies showed that midazolam can be used safely and effectively to induce light or moderate sedation.

Ketamine was administered 12 times in varying concentrations (0.25 mg/kg, 5 mg/kg, 6 mg/kg, 10 mg/kg, and 50 mg/kg) over 9 different studies. It was administered intranasally in 4 studies, intravenously in 2 studies, orally in 5 studies (in one case, the patient was given a ketamine-laced lollipop), and via intramuscular injection in one study. The drug proved to be a highly effective sedative in all of these studies.

Two different studies administered dexmedetomidine intravenously and intranasally in two different concentrations (1 µg/kg and 1.5 µg/kg), and the drug was shown to be a viable alternative to midazolam.

Clonidine was tested in two studies using varying concentrations (2 µg/kg, 4 µg/kg, 3-4 µg/kg, and 7-8 µg/kg). It was administered intranasally twice and orally on one occasion.

Two studies administered atropine intranasally, orally, or intramuscularly in varying concentrations (0.02 mg/kg, 0.05 mg/kg, and 20 mg/kg). Atropine helps to reduce the increased salivation often caused by ketamine, and it also helps facilitate the absorption of clonidine.

Between 1-1.5 mg/kg of propofol were administered intravenously in 2 different articles. Propofol is a safe method of sedation, but it is less potent than intravenous ketamine.

Three articles assessed the use of diazepam in concentrations of 0.5 mg/kg and 5 mg/kg. It was always administered orally, and it proved less effective than midazolam.

Chloral hydrate was administered orally in 2 articles, in concentrations of 40 mg/kg, 50 mg/kg, and 75 mg/kg. The drug provided good results.

The following drugs were used in only one study: 0.5 mg/kg of alprazolam, administered orally; 3 mg of melatonin, also administered orally; 1-1.5 mg/kg of ketofol, administered intravenously; 70 mg/kg of triclofos, administered orally; 0.3 µg/k of fentanyl via submucosal administration; and 2 mg/kg of hydroxyzine. A placebo was administered on six occasions.

## Discussion

There are a wide range of drugs, routes of administration, and varying clinical protocols that can be used to induce conscious or deep sedation.

Benzodiazepines are the class of drugs most often used to induce a state of anxiolysis, sedation, or amnesia (15). Of the articles selected for this review, midazolam is the most frequently used benzodiazepine (16-18-22,23-29).

Midazolam can be used to induce a safe and effective state of sedation without risk of cardiopulmonary complications. This conclusion has been reached after comparing midazolam with other sedative drugs such as diazepam, ketamine, clonidine, and dexmedetomidine in double- and triple-blind randomized studies. In these studies, midazolam provided the best results in terms of onset time of action, depth of sedation, and anxiolysis (16-20,27).

Midazolam can be delivered in various ways, including via intravenous, intramuscular, submucosal, oral, or intranasal routes of administration. The most commonly used routes of administration of midazolam are intranasal, oral, or intravenous. Any of these can induce a state of anxiolysis, but only intravenous administration of midazolam can induce a state of deep sedation, as demonstrated by Tyagi *et al.* in a well-designed prospective randomized triple-blind study (18).

Oral and intramuscular routes of administration result in similar sedative effects, but the former is less invasive and better tolerated by patients, which lends it a significant advantage (24,29). Intranasal administration of a midazolam spray is also an effective method of inducing sedation and fast-onset anxiolysis. A level of moderate sedation can be achieved with this drug and route of administration after about 30 minutes (16,17). However, the spray may cause symptoms such as bitter taste or burning sensations or pain within the nose. These side effects can be avoided by opting for a buccal midazolam spray applied to the oral mucosa, which is well tolerated by uncooperative patients (19,24). Although this method has been studied in various clinical trials, only Klein *et al.* (24) carried out a randomized study.

Midazolam has only been used to sedate children, but it should not be the first option as hypoventilation may occur, depending on dose and any paradoxical reactions (17,29). However, midazolam can be used in conjunction with other sedatives like ketamine or propofol to help decrease the overall dosage needed, which also aids in minimizing any adverse effects and may promote quicker recovery times and a faster onset of sedative action (29). While this was demonstrated by one of the clinical trials evaluated as part of this systematic literature review, there is a need for additional double-blind studies in order to obtain more concrete evidence.

Other diazepines such as diazepam or alprazolam have also been successfully used to sedate patients (15,18,21,30).

Diazepam and midazolam exhibit similar sedative effects, but the latter provides a better anxiolytic effect as well as a minimally higher level of sedation; therefore, diazepam does not offer any sedative advantage over midazolam (18,21). Alprazolam is a highly effective anxiolytic premedication, and when combined with melatonin it increases the latter’s sedative effects, thus inducing a deep level of sedation.

Ketamine is a dissociative anesthetic and analgesic that is also used as a sedative drug, maintaining the patient’s muscle tone and the respiratory system’s protective reflexes (29,31). However, in adults, ketamine may also cause hallucinations and nightmares during the recovery period, and as such it sees limited use in adults; these side effects are rarely seen in children (31).

Intravenous ketamine has been shown to have a powerful sedative effect; some researchers actually preferred ketamine to midazolam due to increased patient cooperativeness and because it carried less side effects; more double- and triple-blind studies are necessary to compare its effectiveness with that of other drugs in order to obtain sufficient scientific evidence for this claim (32).

On the other hand, when comparing oral midazolam and oral ketamine, while they exhibit similar sedative effects, midazolam is more conducive to anxiolysis, and orally administered ketamine results in a slower recovery period post-sedation. These drugs were compared in a well-designed, double-blind randomized clinical trial (27).

Ketamine can be delivered safely and effectively via an intranasal route of administration (16,26,29).

Transmucosal oral administration of ketamine has also been studied using a lollipop to deliver the drug, and its effectiveness was then compared with oral midazolam without evidence of any greater sedative effects. However, only one of the studies reviewed examined this route of administration of ketamine, and while it was a randomized study, there is a need for additional, double-blind studies in order to obtain better evidence to this effect (31).

A combination of oral ketamine and oral midazolam results in safe and effective sedation (22,23,29), and a combination of oral ketamine, oral midazolam and atropine significantly reduces the increased salivation often caused by ketamine (29). However, unfortunately these were not double-blind studies, which would have provided more concrete evidence.

Propofol is a short-acting intravenous sedative. This drug is very useful for surgical interventions in the orofacial area, which necessitate a higher quantity of local anesthesia with adrenaline; propofol can help balance cardiovascular alterations resulting from the adrenaline injection (32).

The sedative effects of propofol are stronger than those of midazolam, but it can also cause additional side effects such as sudden movements, crying fits, intermittent coughing, and pain at the site of injection. Additionally, there is a risk of severe hypotension when administering propofol (33). However, while these studies were randomized, they would benefit from larger sample sizes and a double-blind design in order to provide better evidence of this.

Propofol can also be combined with ketamine, although the combination of both these drugs may lead to hypoventilation. Therefore, as both drugs have similar sedative effects, it is safer to use propofol alone rather than combining the two sedatives. This is seen in Mittal *et al.’s* study; although a randomized double-blind clinical trial, the study would have benefitted from a larger sample size (34).

Clonidine and dexmedetomidine target the α-2 adrenergic receptor agonists, and their potential use as preoperative premedications has been studied extensively.

Intranasal clonidine appears to induce an adequate level of sedation, working more slowly than midazolam but with fewer side effects, as demonstrated in a rigorous, randomized double-blind study with a good sample size (17). Additionally, when combined with atropine, clonidine appears to be better absorbed and to have a more predictable effect on the reduction of nasal secretions (17).

Other studies found that clonidine did not have very strong sedative effects (35). Clonidine has also been studied in conjunction with ketamine and midazolam, without any additional benefits being found from using this combination (22).

Despite this, dexmedetomidine appears to function just as well as midazolam, providing a safe, moderate, and effective sedation. Additionally, some studies suggest it may even increase patient cooperation (16,20).

Chloral hydrate is another safe and effective sedative drug, but it does not appear to have any advantages over midazolam (28,30). A combination of chloral hydrate and hydroxyzine only results in more side effects, and therefore this combination should not be administered to patients (28). Triclofos was shown to have a less powerful sedative effect than midazolam (25). A combination of midazolam with submucosally administered opioids such as fentanyl results in a greater sedative effect, but this may also cause oxygen desaturation (26).

In conclusion, Midazolam is the most commonly used sedative drug in dental procedures (light sedation). It is a very safe sedative, and it is most often administered either intranasally or orally.

Ketamine also proves very useful when administered intranasally, inducing a high level of sedation (deeper than that of midazolam); however, when delivered via other routes of administration, various side effects have been observed. Intravenous propofol is also a very safe and effective sedative.

Other drugs like clonidine and dexmedetomidine have also been proven effective in inducing a state of conscious sedation.

However, further clinical trials are needed to compare these drugs and obtain more evidence in order to determine which of these are the safest and most effective.
